# Biological nomenclature terms for facilitating communication in the naming of organisms

**DOI:** 10.3897/zookeys.192.3347

**Published:** 2012-05-08

**Authors:** John David, George M. Garrity, Werner Greuter, David L. Hawksworth, Regine Jahn, Paul M Kirk, John McNeill, Ellinor Michel, Sandra Knapp, David J. Patterson, Brian J. Tindall, Jonathan A. Todd, Jan van Tol, Nicholas J. Turland

**Affiliations:** 1 Royal Horticultural Society Garden Wisley, Woking, Surrey, GU23 6QB, UK; 2 Microbiology and Molecular Genetics, Michigan State University, Biomedical and Physical Sciences, 567 Wilson Road Room 6162, East Lansing, MI 48824-4320 USA; 3 Botanischer Garten und Botanisches Museum Berlin-Dahlem, Freie Universität Berlin, Königin-Luise-Straße 6-8, D-14195 Berlin, Germany; and Herbarium Mediterraneum, c/o Orto Botanico, via Lincoln 2/A, I-90121 Palermo, Italy; 4 Departamento de Biología Vegetal II, Facultad de Farmácia, Universidad Complutense de Madrid, Plaza Ramón y Cajal, 28040 Madrid, Spain; & Department of Life Sciences, The Natural History Museum, Cromwell Road, London SW7 5BD, UK; 5 Botanischer Garten und Botanisches Museum Berlin-Dahlem, Freie Universität Berlin, Königin-Luise-Straße 6-8, D-14195 Berlin, Germany; 6 CABI UK, Bakeham Lane, Egham, Surrey TW20 9TY, UK; 7 Royal Botanic Garden, Edinburgh, EH3 5LR, Scotland, UK; and Royal Ontario Museum, Toronto, Canada; 8 International Trust for Zoological Nomenclature, The Natural History Museum, Cromwell Road, London SW7 5BD, UK; 9 Department of Life Sciences, The Natural History Museum, Cromwell Road, London SW7 5BD, UK; 10 Marine Biological Laboratory, 7 MBL Street, Woods Hole MA 02543, USA; 11 Leibniz-Institut DSMZ-Deutsche Sammlung von Mikroorganismen und Zellkulturen GmbH, Inhoffenstraße 7B, 38124 Braunschweig, Germany; 12 Department of Earth Sciences, The Natural History Museum, Cromwell Road, London SW7 5BD, UK; 13 Leiden University, National Museum of Natural History (Naturalis), P.O. Box 9517, NL-2300 RA Leiden, The Netherlands; 14 Missouri Botanical Garden, P.O. Box 299, St. Louis, MO 63166-0299, USA

**Keywords:** Nomenclature, Code, terminology

## Abstract

A set of terms recommended for use in facilitating communication in biological nomenclature is presented as a table showing broadly equivalent terms used in the traditional *Codes* of nomenclature. These terms are intended to help those engaged in naming across organism groups, and are the result of the work of the International Committee on Bionomenclature, whose aim is to promote harmonisation and communication amongst those naming life on Earth.

The International Committee on Bionomenclature (ICB, http://www.bionomenclature.net/) met in Berlin from 26–28 April 2012. As a part of this meeting it reviewed the status of communication between and change in the various international sets of rules that biologists follow when naming organisms – the *Codes* of nomenclature. The group exchanged updates on the status of the *Codes* (see Table 1 for abbreviations used for the various *Codes* of nomenclature) and discussed how to enhance inter-community communication with the aim of bringing together those concerned with naming life on Earth.

Recent progress on developing a Global Names Architecture (http://www.globalnames.org) has meant that the communities working on the various indices for a variety of organism groups are not only working in their own domains, but are increasingly developing technological solutions to enable more efficient retrieval of names of all organisms, along with information pertaining to their first publication. As groups focused on the nomenclature of various organisms work more closely together, efficient communication becomes ever more important. Recent changes in the rules governing the naming of prokaryotes (Labeda 2000; and for example Labeda and Oren 2011) and of algae, fungi and plants (see Hawksworth 2011; Knapp et al. 2011; McNeill and Turland 2011), in addition to those proposed for zoology (e.g., International Commission on Zoological Nomenclature 2008), are bringing the terminology used in and practices of the *Codes* closer together, and the Committee felt that agreement on a basic set of terms to be used when engaging in inter-community communication would greatly assist this on-going process. Naming of organisms is so critical that it is important that we work together on a greater consistency in nomenclatural practices to enable a swifter, more efficient documentation of biodiversity and help meet the global challenges of understanding Earth’s genetic diversity and resources.

**Table 1.** Recommended terms for use in biological nomenclature with a comparison of equivalents across six current *Codes* of nomenclature

**Bionomenclature**	**ICN^1^**	**ICNCP^2^**	**ICNP^3^**	**ICVCN^4^**	**ICZN^5^**	**PhyloCode^6^**
*Publication and precedence of names*						
published	effectively published	published	effectively published	[none]	published	published
precedence/priority	priority	priority	priority	[none]	precedence/priority	precedence
earlier	earlier	earlier	earlier	[none]	senior	earlier
later	later	later	later	[none]	junior	later
*Nomenclatural status*						
established	validly published	established	validly published	established	available	established
compliant	legitimate	acceptable	legitimate	valid	potentially valid	acceptable
non-compliant	illegitimate	[none]	illegitimate	[none]	permanently invalid	[none]
registered	[deposited]	registered	validly published	[none]	registered	registered
*Taxonomic status*						
accepted	correct	accepted	correct	accepted	valid	accepted
*Synonymy and homonymy*						
homotypic	homotypic	[none]	homotypic	[none]	objective	homodefinitional
heterotypic	heterotypic	[none]	heterotypic	[none]	subjective	heterodefinitional
replacement name	replacement name	replacement name	replacement name	[none]	new replacement	replacement name
*Conservation and suppression*						
conserved	conserved	conserved	conserved	[none]	conserved	conserved
protected	listed	[none]	listed^7^	accepted	protected	[none]
sanctioned (fungi only)	sanctioned (fungi only)	[none]	[none]	[none]	[none]	[none]
suppressed/rejected	rejected	rejected	rejected	[none]	suppressed	suppressed
*Types of names*						
name-bearing type	nomenclatural type	nomenclatural standard	nomenclatural type	[none]	name-bearing type	[none]
nominal taxon	name and type	[none]	name and type	[none]	nominal taxon	[none]

^1^ *International Code of Nomenclature for algae, fungi, and plants* (ICN) or *Melbourne Code* (McNeill et al. 2012). It is expected to be available online in 2013 at http://www.iapt-taxon.org ^2^ *International Code of Nomenclature for Cultivated Plants* (ICNCP) or *Cultivated Plant Code*, 8th edition (Brickell et al. 2009); http://www.actahort.org/chronica/pdf/sh_10.pdf ^3^ *International Code of Nomenclature of Prokaryotes* (the name adopted for the *International Code of Nomenclature of Bacteria* (ICNB) or *Bacteriological Code* (Lapage et al. 1992), see Labeda 2000): http://www.ncbi.nlm.nih.gov/books/NBK8817/) ^4^ *The International Code of Virus Classification and Nomenclature* (ICVCN), in Virus Taxonomy (ed. King et al. 2011) ^5^ *International Code of Zoological Nomenclature* (ICZN), 4th edition (International Commission on Zoological Nomenclature 1999): http://www.nhm.ac.uk/hosted-sites/iczn/code/ ^6^ *International Code of Phylogenetic Nomenclature* or *PhyloCode*, version 4c (Cantino and Queiroz 2010): http://www.ohio.edu/phylocode/ ^7^ Listed in the sense of appearing on *The Approved Lists of Bacterial Names*

This table of terms is not comprehensive, but includes those terms that differ (or have differed in the past) significantly and have the potential to cause confusion. It is based on the table of equivalence of technical terms arising from discussions on harmonisation of nomenclature (Hawksworth 1995) and that accompanying the first *Draft BioCode* (Greuter et al. 1996). These early attempts have here been updated to reflect current usage of terms in the various *Codes*. As with the early tables, the terms in each row are not perfectly congruent. We recommend the use of these terms to facilitate communication between those working with the nomenclature of different groups of organisms without necessarily displacing those used by tradition within the various communities. These terms can be employed where considered of value in presentations, publications, and teaching, as well as in discussions between the communities who use the different *Codes*. We invite and welcome comment on the commended terms, and suggestions for other terms that have caused confusion that might be added – our aim is not to impose practice, but to facilitate communication among all involved in the naming of organisms of all kinds.

## Current membership of the International Committee on Bionomenclature

(ICB – contact details for all members are available at http://www.bionomenclature.net/)

Michael J. Adams, Rothamsted, UK

Philip D. Cantino, Athens Ohio, USA

John David, Wisley, Surrey, UK

George Garrity, East Lansing, Michigan, USA

Daphne Fautin, Lawrence, Kansas, USA

Werner Greuter, Berlin, Germany and Palermo, Italy

David L. Hawksworth, London, UK and Madrid, Spain

Regine Jahn, Berlin, Germany (Deputy Secretary)

Paul M Kirk, Egham, Surrey, UK

John McNeill, Edinburgh, Scotland, UK and Toronto, Canada

Ellinor Michel, London, UK (Vice-Chair)

Sandra Knapp, London, UK (Chair)

David Patterson; Woods Hole, Massachusetts, USA (Secretary)

Richard Pyle, Honolulu, Hawa’i, USA

David Remsen, Copenhagen, Denmark

Gary Rosenberg, Philadelphia, Pennsylvania, USA

Brian Tindall, Braunschweig, Germany

Jonathan A. Todd, London, UK

Jan van Tol, Leiden, The Netherlands

Nick Turland, St. Louis, Missouri, USA

## References

[B1] Brickell CD Alexander C David JC Hetterscheid WLA Leslie AC Malecot V Jin XB Cubey JJ (2009) International Code of Nomenclature for Cultivated Plants (ICNCP or Cultivated Plant Code) incorporating the Rules and Recommendations for naming plants in cultivation. Eighth Edition. Adopted by the International Union of Biological Sciences International Commission for the Nomenclature of Cultivated Plants. Regnum Vegetabile 151; Scripta Horticulturae 10. Leuven: International Society for Horticultural Science.

[B2] Cantino PD Queiroz K de (2010) International Code of Phylogenetic Nomenclature, version 4c. http://www.ohio.edu/phylocode/.

[B3] Greuter WG Hawksworth DL McNeill J Mayo MA Minelli A Sneath PH Tindall BJ Trehane P Tubbs P (1996) Draft BioCode: the prospective international rules for the scientific names of organisms. Taxon 45: 349-373

[B4] Hawksworth DL (1995) Steps along the road to a harmonized bionomenclature. Taxon 44: 447-456

[B5] Hawksworth DL (2011) A new dawn for the naming of fungi: impacts of decisions made in Melbourne in July 2011 on the future regulation and publication of fungal names. MycoKeys 1: 7-20 doi: 10.3897/mycokeys.1.2062 10.5598/imafungus.2011.02.02.06 PMC3359813 22679600

[B6] International Commission on Zoological Nomenclature (1999) International Code of Zoological Nomenclature, fourth edition, Ride WDL, Cogger HG, Dupuis C, Kraus O, Minelli A, Thompson FC, Tubbs PK (eds.). Adopted by the International Union of Biological Sciences. London: International Trust for Zoological Nomenclature.

[B7] International Commission on Zoological Nomenclature (2008) Proposed amendment of the *International Code of Zoological Nomenclature* to expand and refine methods of publication. Zootaxa 1908: 57-67

[B8] International Committee on Taxonomy of Viruses (2011) The International Code of Virus Classification and Nomenclature of ICTV. Pp. 1273–1277 In: King AMQ, Lefkowitz EJ, Adams MJ, Carstens EB (Eds) Virus taxonomy: ninth report of the International Committee on Taxonomy of Viruses. Amsterdam: Elsevier Academic Press.

[B9] Knapp S McNeill J Turland N (2011) Changes to publication requirements made at the XVIII International Botanical Congress in Melbourne - what does e-publication mean for you? PhytoKeys 6: 5–11. doi: 10.3897/phytokeys.6.1960 10.3897/phytokeys.6.1960 PMC3261035 22287918

[B10] Labeda DP (2000) International Committee on Systematic Bacteriology. IXth International (IUMS) Congress of Bacteriology and Applied Microbiology. Minutes of the meetings, 14 and 17 August 1999, Sydney, Australia. International Journal of Systematic and Evolutionary Microbiology 50: 2245–2247. http://ijs.sgmjournals.org/content/50/6/2245.full.pdf+html 10.1099/00207713-50-3-1405 10843089

[B11] Labeda DP Oren A (2011) International Committee on Systematics of ProkaryotesXIIth International (IUMS) Congress of Microbiology and Applied Bacteriology Minutes of the Meetings, 3, 4, 5 and 7 August 2008, Istanbul, Turkey. International Journal of Systematic and Evolutionary Microbiology 61: 2781-2789

[B12] Lapage SP Sneath PHA Lessel EF Skerman VDB Seeloger HPR Clark WA (Eds) (1992) International Code of Nomenclature of Bacteria and Statutes of the International Committee on Systematic Bacteriology and Statutes of the Bacteriology and Applied Microbiology Section of the International Union of Microbiological Societies. Bacteriological Code (1990 Revision). Washington, DC: American Society for Microbiology.

[B13] McNeill J Barrie FR Buck WR Demoulin V Greuter W Hawksworth DL Herendeen PS Knapp S Marhold K Prado J Prud’homme van Reine WF Smith GF Wiersema JH Turland NJ (Eds) (2012, in press) International Code of Nomenclature for algae, fungi, and plants (Melbourne Code). Adopted by the Eighteenth International Botanical Congress Melbourne, Australia, July 2011. Regnum Vegetabile. Ruggell: Gantner.

[B14] McNeill J Turland N (2011) Major changes to the Code of Nomenclature – Melbourne, July 2011. Taxon 60: 1495-1497

